# Post-therapy emergence of an *NBN* reversion mutation in a patient with pancreatic acinar cell carcinoma

**DOI:** 10.1038/s41698-024-00497-x

**Published:** 2024-04-01

**Authors:** Meredith S. Pelster, Ian M. Silverman, Joseph D. Schonhoft, Adrienne Johnson, Pier Selenica, Danielle Ulanet, Victoria Rimkunas, Jorge S. Reis-Filho

**Affiliations:** 1 Sarah Cannon Research Institute/Tennessee Oncology, Nashville, TN USA; 2 Repare Therapeutics, Cambridge, MA USA; 3 Department of Pathology, Memorial Sloan Kettering Cancer Center, New York, NY USA

**Keywords:** Pancreatic cancer, Cancer genomics, Targeted therapies, Genomic instability, DNA damage response

## Abstract

Pancreatic acinar cell carcinoma (PACC) is a rare form of pancreatic cancer that commonly harbors targetable alterations, including activating fusions in the MAPK pathway and loss-of-function (LOF) alterations in DNA damage response/homologous recombination DNA repair-related genes. Here, we describe a patient with PACC harboring both somatic biallelic LOF of *NBN* and an activating *NTRK1* fusion. Upon disease progression following 13 months of treatment with folinic acid, fluorouracil, irinotecan, and oxaliplatin (FOLFIRINOX), genomic analysis of a metastatic liver biopsy revealed the emergence of a novel reversion mutation restoring the reading frame of *NBN*. To our knowledge, genomic reversion of *NBN* has not been previously reported as a resistance mechanism in any tumor type. The patient was treated with, but did not respond to, targeted treatment with a selective NTRK inhibitor. This case highlights the complex but highly actionable genomic landscape of PACC and underlines the value of genomic profiling of rare tumor types such as PACC.

## Introduction

Pancreatic acinar cell carcinomas (PACCs) are rare forms of pancreatic cancer, accounting for approximately 1–2% of pancreatic tumors in adults and 15% of those in children^1^. PACCs are more prevalent in men, with a male to female ratio of 2:1. Histologically, these neoplasms are characterized by tumor cells displaying features of acinar cell differentiation, with moderate amounts of eosinophilic cytoplasm containing periodic acid-Schiff (PAS)-positive diastase-resistant zymogen granules and uniform nuclei with distinct nucleoli, arranged in acinar, glandular, solid, or trabecular patterns^1^. Genomically, PACCs have a repertoire of alterations distinct from that of pancreatic ductal carcinomas, rarely displaying *KRAS*, *TP53, CDKN2A*, and *SMAD4* somatic alterations, and more frequently harboring recurrent activating fusions affecting *BRAF, RET*, *RAF1*, and *NTRK1/2/3*, which are present in up to 30% of patients^2–7^. In addition, a subset of patients with PACCs harbor germline pathogenic alterations affecting DNA damage response (DDR)/homologous recombination (HR) DNA repair-related genes, including *ATM*, *BRCA1*, *BRCA2*, and *PALB2*^2,3,8^.

The mainstay of treatment for patients with PACCs is surgical resection with negative margins followed by chemotherapy. Currently, no targeted therapies are approved by the US Food and Drug Administration specifically for patients with PACC. Patients whose tumors carry oncogenic fusions may be treated using therapies with tumor-agnostic labels, such as entrectinib or larotrectinib for NTRK fusions or selpercatinib for RET fusions^9–11^. Inhibitors of poly (ADP-ribose) polymerase (PARP) have been approved for patients with pancreatic cancer (including PACC) harboring germline *BRCA1* or *BRCA2* pathogenic mutations, and further studies are investigating the benefit of PARP inhibitors in pancreatic cancers with HR deficiency (HRD) due to alterations affecting other canonical HR-related genes (NCT04858334), and in unselected PACC (NCT05286827).

The nibrin gene (*NBN*, also known as *NBS1* [Nijmegen breakage syndrome 1]) is an integral component of the DDR/HR DNA repair machinery^12,13^. Germline *NBN* loss-of-function (LOF) alterations have not only been causally implicated in the Nijmegen breakage syndrome, but have also been shown to result in an increased risk of multiple cancer types^13^. The impact of somatic pathogenic *NBN* alterations on the development and progression of pancreatic cancer development is uncertain.

In this report, we describe a patient with PACC whose tumor harbored both an activating *SEL1L::NTRK1* fusion and somatic biallelic inactivation of *NBN*. Upon disease progression on treatment with folinic acid, fluorouracil, irinotecan, and oxaliplatin (FOLFIRINOX), a novel reversion mutation restoring the *NBN* reading frame was detected, suggesting a potential link between *NBN* LOF, PACC progression, and the evolution of therapeutic resistance to mainstay treatment. This novel reversion mutation affecting the *NBN* gene adds to the growing list of resistance mechanisms to DNA damaging- and DDR-targeted therapies.

## Results

### Clinical case

**Fig. 1:**
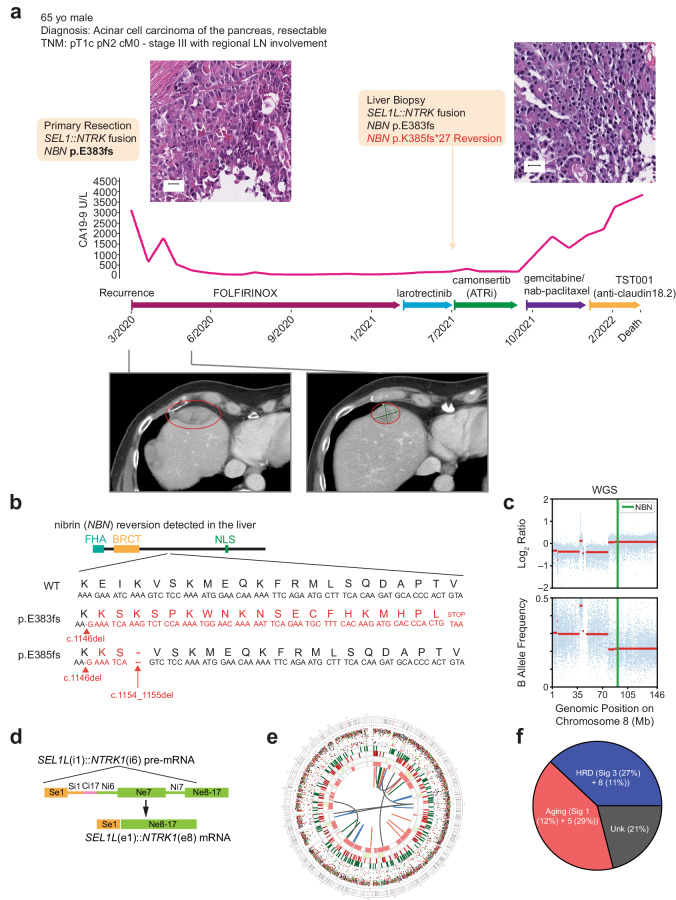
Case study of a 65-year-old male diagnosed with pancreatic cancer who developed a reversion mutation in *NBN* after more than 1 year on FOLFIRINOX. **a** Therapy history and CA19-9 kinetics. Representative micrographs of the primary resection and metastatic liver biopsy specimens revealed the presence of acinar structures comprising neoplastic cells with eosinophilic and granular cytoplasm, and atypical nuclei, consistent with a diagnosis of pure acinar cell carcinoma of the pancreas (scale bar: 20 µm). Representative CT scans pre- and on-treatment with FOLFIRINOX. **b** *NBN* reversion mutation detected in the metastatic liver biopsy. The reference (top), primary mutation (middle), and secondary mutation (bottom) cDNA and amino acid sequences are shown. The combination of the primary and secondary mutations created a 3-amino acid deletion and 2-amino acid insertion resulting in a novel in-frame indel (c.1146_1155delinsGAAATCA; p.383_385delinsKS). **c** B allele frequency/log R ratio plots derived from WGS analysis of the metastatic liver biopsy. **d** Genomic and transcriptomic structure of the *SEL1L::NTRK1* fusion. Skipping of *NTRK1* exon 7 was observed in the targeted RNA sequencing data, reflective of the coding potential of different splice configurations. **e** Chromosome-based circos plot derived from WGS of the metastatic liver biopsy. **f** Distribution of mutational signatures as determined by WGS of the metastatic liver biopsy. CA cancer antigen, cDNA complementary DNA, CT computed tomography, FOLFIRINOX folinic acid, fluorouracil, irinotecan, and oxaliplatin, indel insertion and deletion, Mb megabase, WGS whole genome sequencing.

A 65-year-old White male patient presented with abdominal pain. Computed tomography showed a mild distension of the common bile duct, and further evaluation with an endoscopic ultrasound revealed a mass in the pancreatic head. Fine needle aspiration of this mass was performed, and cytology was consistent with adenocarcinoma (Fig. 1a). Staging evaluation indicated pT1c, pTN2, pM0 (AJCC 8^th^ edition^14^), and the patient underwent pancreaticoduodenectomy. Pathologic examination revealed an acinar cell carcinoma measuring 2 cm with varied architecture, including solid trabecular and acinar formations that invaded the duodenum and intrahepatic common bile duct, with 4 of 16 regional lymph nodes involved. At the cellular level, cytoplasms were granular with monomorphic nuclei containing prominent nucleolei. Consistent with the diagnosis, immunohistochemical analysis were negative for synaptophysin, chromogranin, and MOC-31/EpCAM, while periodic acid-Schiff staining with diastase digestion (PAS-D) were positive for cytoplasmic granules. Given the rarity of the tumor type, the diagnosis was confirmed and agreed upon by multiple pathologists. Furthermore, the screening tissue used to determine molecular eligibility was retrospectively found to be BCL-10 positive consistent with the diagnosis^1^.

Subsequently, the patient received adjuvant treatment with gemcitabine and capecitabine for 6 months, followed by radiation concurrent with capecitabine. After completion of this treatment, the patient was on surveillance for 16 months, at which time he was diagnosed with metastatic disease to the liver, histopathologically confirmed as metastatic acinar cell carcinoma (Fig. 1a).

At that time, a tissue specimen from the primary pancreaticoduodenectomy (herein referred to as the primary resection) was sent for clinical-grade next generation sequencing (NGS) as per standard of care, which identified a somatic pathogenic *NBN* mutation and an in-frame *SEL1L::NTRK1* fusion (Table 1). The patient then received palliative chemotherapy with the modified FOLFIRINOX regimen for 13 months. The patient achieved an initial response with a marked decrease in cancer antigen (CA)19-9 and a reduction in hepatic tumor burden, but treatment was eventually discontinued due to the development of a new liver lesion and an increase in the size of prior liver lesions (Fig. 1a). Given the presence of an NTRK1 fusion in the primary resection specimen, the patient received targeted treatment with the NTRK inhibitor larotrectinib. However, the patient rapidly progressed with multiple new liver lesions and an increase in the size of prior liver lesions, resulting in treatment discontinuation 2 months later.

**Table 1:** Summary of genomic alterations identified

Specimen	Assay	*NBN* alterations (VAF)	*NBN* allelic status	Rearrangements/fusion	Other alterations	Genomic signatures
Primary resection (April 2018)	Tempus xT (DNA/RNA)	p.E383Kfs*21 (54.6%)	–	*SEL1L::NTRK1* fusion	*RB1* p.Q217* (7.6%), *CKS1B* gain	TMB = 2.1, MSS
	SNiPDx™	p.E383Kfs*21 (29%)	Biallelic (CN LOH)	–	None detected	–
	Archer FusionPlex	–	–	*SEL1L*(e1)*::NTRK1*(e8) fusion	–	–
Metastatic liver biopsy (July 2021)	FoundationOne CDx	p.E383Kfs*21 (73.5%), p.K385fs*27 (33.4%)	–	None detected	*MCL1* Amp	TMB = 3.0, MSS
	SNiPDx™	p.E383Kfs*21 (55%), p.K385fs*27 (28%)	Reverted (CN LOH)	–	*RB1* c.2326-20_2326-2del (42%)	–
	WGS	p.E383Kfs*21 (66%), p.K385fs*27 (38%)	Reverted (CN LOH)	*SEL1L*(i1)*::NTRK1*(i6) rearrangement	*MCL1* Amp, *RB1* c.2326-20_2326-2del (36%), *SMAD4* HomDel	TMB = 3.0, MSS, SBS = 1/5/3/8, HRDetect negative

*Amp* amplification, *CN LOH* copy-neutral loss of heterozygosity, *e* exon, *HomDel* homozygous deletion, *i* intron, *MSS* microsatellite stable, *SBS* single base substitution, *SNiPDx* Synthetic Lethal Interactions for Precision Diagnostics, *TMB* tumor mutational burden, *VAF* variant allele frequency, *WGS* whole genome sequencing.

A new liver biopsy (herein referred to as the metastatic liver biopsy) was taken at this time and sent for clinical-grade NGS (Table 1). Pending the results and based on the detection of a pathogenic *NBN* mutation in the primary resection specimen, the patient was enrolled on TRESR (NCT04497116) and received a therapeutic dose level of the ataxia telangiectasia and Rad3-related (ATR) inhibitor camonsertib (RP-3500, Fig. 1a). The patient had stable disease at 6 weeks, but treatment was discontinued after 12 weeks due to progression of the target liver lesions (32% increase). The patient was then treated with gemcitabine and paclitaxel (nab-paclitaxel was not available due to a nationwide shortage), on which he also experienced progression of disease after 2 months due to worsening peritoneal carcinomatosis. Subsequently, he was briefly treated on a clinical trial with TST001, an anti-claudin 18.2 monoclonal antibody, before discontinuing the study due to clinical progression after which the patient was enrolled in a hospice and passed away 1 month later.

### Genomic analysis

Genomic analyses of the tissue specimens obtained during the course of treatment were performed with clinical-grade NGS assays, as well as with research-use-only NGS assays performed as part of TRESR (NCT04497116; Table 1). This included the use of the Synthetic Lethal Interactions for Precision Diagnostics (SNiPDx™) panel, a novel NGS-based diagnostic panel specifically designed to detect and distinguish between monoallelic and biallelic LOF alterations in genes determined to be synthetic lethal with camonsertib^15^. The primary resection specimen collected 3 months after diagnosis was initially evaluated by clinical-grade NGS and retrospectively by SNiPDx™, and anchored multiplex polymerase chain reaction (PCR) (AMP)-based RNA sequencing (Table 1)^15–18^. The metastatic liver biopsy specimen, collected after treatment with FOLFIRINOX and larotrectinib and just prior to initiation of camonsertib, was initially evaluated by clinical-grade NGS, and retrospectively by SNiPDx™ and whole genome sequencing (WGS)^15,19–21^.

DNA analysis of both tissue specimens and peripheral blood mononuclear cells (PBMCs) by multiple methodologies identified a somatic (absent in PBMCs) pathogenic 1-nucleotide deletion in exon 10 of *NBN* (c.1146del; p.E383Kfs*21; ClinVar Variation ID: 822251; Table 1 and Fig. 1b). This deletion results in a premature stop codon 21 codons downstream of p.E383 and is predicted to truncate the protein leading to nonsense-mediated decay. Analysis of the allele-specific copy number by SNiPDx™ and WGS revealed copy-neutral loss of heterozygosity of the *NBN* wild-type allele with subsequent duplication of the mutant allele, consistent with a clonal somatic biallelic inactivation of *NBN* (Fig. 1c and Supplementary Fig. 1). In addition, a subclonal *RB1* (c.649 C > T; p.Q217*) pathogenic mutation was detected in the primary tumor resection. No germline or somatic pathogenic alterations affecting *TP53, ATM, BRCA1, BRCA2, PALB2*, or other DDR/HR-related genes included in the assays were detected. No *RET*, *BRAF*, or *RAF1* fusions were detected by WGS, targeted DNA sequencing or targeted RNA sequencing; however, we detected a complex structural rearrangement involving exon 1 of *SEL1L* and exon 8 of *NTRK1* resulting in an expressed, in-frame, likely oncogenic *SEL1L::NTRK1* fusion containing the NTRK1 kinase domain (Table 1 and Fig. 1d, e).

### Novel *NBN* reversion mutation in the metastatic biopsy

A genomic comparison of the biopsies from the liver metastasis obtained upon progression on FOLFIRINOX and the primary tumor resection revealed a novel *SMAD4* homozygous deletion, an *MCL1* amplification, and a second frameshift mutation in *NBN*. This *NBN* mutation, which was detected only in the liver metastasis specimen, but absent in the primary resection specimen, resulted in an additional 2-nucleotide deletion (c.1154_1155del; p.K385fs*27) 8 nucleotides downstream of the initial somatic mutation (Table 1 and Fig. 1b). Visual inspection of the genomic sequencing reads revealed that the *NBN* mutations were in *cis* (data not shown) and predicted to re-establish the *NBN* coding frame.

### Multimodal detection of NTRK1 rearrangement and fusion

Following progression on FOLRIFINOX, the patient received larotrectinib due to the presence of an NTRK1 fusion. The patient, however, rapidly progressed within 2 months of initiating therapy. Given the lack of response to NTRK-targeted therapy, we aimed to validate the presence of the NTRK1 fusion. Targeted RNA sequencing of the primary resection confirmed the presence of a *SEL1L::NTRK1* fusion and further delineated the breakpoints for the fusion transcript to be at exon 1 of *SEL1L* and exon 8 of *NTRK1*, resulting in an in-frame fusion containing the NTRK kinase domain. To understand the genomic mechanisms underlying this rare molecular anomaly, we examined the genomic rearrangements identified by WGS of the metastatic liver biopsy. No direct interaction was identified between *SEL1L* and *NTRK1*, rather, both genes rearranged with the same intron of *CENPC* (intron 17) resulting in a “bridged fusion” between *SEL1L* intron 1 and *NTRK1* intron 6 (Fig. 1d, e)^22^. These data demonstrate that the skipping of *NTRK1* exon 7 and usage of the next proximal splice site, exon 8, led to productive expression of the fusion transcript. Thus, the combined analysis of targeted RNA sequencing and WGS elucidated both the genomic and post-transcriptional details of this rare molecular event. The lack of larotrectinib antitumor activity in this case cannot be explained by the absence of an in-frame NTRK1 fusion, suggesting that another mechanism of primary resistance may be involved.

### Characterization of genomic signatures

To identify additional genomic features of the tumor, we performed WGS on the metastatic liver biopsy specimen only, as the available primary resection specimen had insufficient tumor fraction and residual DNA. This analysis revealed the presence of 8789 single nucleotide variants (SNVs), 319 insertions and deletions (indels; 161 insertions and 158 deletions), and 37 structural variants (16 translocations, 8 deletions, 8 inversions, and 5 tandem duplications; Fig. 1e). The tumor specimen was found to be microsatellite-stable with a tumor mutational burden of 3.0 mutations per megabase, and an estimated tumor ploidy of 1.7. The dominant mutational signatures were related to aging (signatures 1 [12%] + 5 [29%]), followed by HRD (signatures 3 [27%] + 8 [11%]; Fig. 1f). The tumor, however, displayed only a partial set of the genomic features consistent with HRD. Despite the dominant HRD-related single nucleotide substitution signature and genome-wide loss-of-heterozygosity patterns, the enrichment for deletions with microhomology was modest, and rearrangement signatures associated with HRD were not observed. Consistent with these findings, the HRDetect score was 0.24, well below the cutoff used to classify a tumor as HRD (0.7). These results are consistent with genomic scars present in cancers with LOF alterations in genes upstream in the DDR pathway^23–25^.

## Discussion

PACCs have been shown to harbor DDR/HR DNA repair defects, but somatic loss of *NBN* has not been linked to the development of pancreatic cancers in general, or specifically to PACC. In this case study, we performed detailed multimodal genomic profiling of multiple biopsies from the same patient with PACC revealing a comprehensive genomic characterization of the tumor evolution during multiple lines of therapy.

Our findings provide circumstantial evidence to suggest that the somatic *NBN* biallelic mutation likely played a role in the development and/or progression of this PACC. First, the biallelic inactivation of *NBN* through somatic mutation and copy-neutral loss of heterozygosity was linked to genomic signatures consistent with a partial HRD profile, but lacking long indels with (micro)homology, the main driver of HRDetect scores. This is expected, given that HRDetect was trained on genomes from patients with *BRCA1/BRCA2* LOF in breast cancer, whereas NBN plays a role further upstream of BRCA1/BRCA2 in the repair of DNA double-strand breaks^12,23^. Although the genomic “scars” present in canonical HR-related genes (eg, *BRCA1, BRCA2, PALB2, RAD51C, RAD51D*, and *RAD51B*) and their respective absence in cancers with alterations affecting DDR-related genes (eg, *ATM* and *CHEK2*) have been described, the spectrum of genomic signatures in tumors with LOF of less common HR/DDR genes, including *NBN*, has yet to be fully characterized. Our findings emphasize the importance of further studies characterizing the genomic scars stemming from LOF of specific HR/DDR genes, which may not only provide mechanistic insights about back-up DNA repair mechanisms operating in absence of these genes, but also novel therapeutic opportunities based on synthetic lethality approaches^26^.

Second, an additional alteration predicted to restore the *NBN* reading frame was detected following initial treatment and therapeutic vulnerability to FOLFIRINOX. We interpreted this to be a novel reversion mutation based on (1) the potential selective pressure of the platinum-containing regimen FOLFIRINOX; (2) the biallelic nature of the primary *NBN* mutation in the tumor; (3) the proximity; and (4) the *cis* nature of the second *NBN* mutation to the first; and (5) the potential to re-establish the coding frame in the absence of an intervening stop codon. Identification of an emergent *NBN* reversion mutation in the post-therapy specimen might suggest an adaptive resistance mechanism mediated by restoration of NBN function. Reversion alterations in *BRCA1/2, PALB2, RAD1C*, and *RAD51D* have been documented as mechanisms of resistance to platinum-containing therapies and PARP inhibitors^27^. To our knowledge, this is the first report of a reversion in *NBN* following treatment with platinum or PARP inhibitor therapy in any tumor type.

Third, the patient did not respond to a selective NTRK inhibitor despite demonstrated expression of an in-frame *NTRK1* fusion containing the kinase domain. This was unexpected, given the high response rates for NTRK inhibitors across tumor types including pancreatic cancers^9,10^. Most notably, a recent case report described a patient with PACC with the same *SEL1L::NTRK1* fusion reported in this study, but lacking any other likely driver alterations, who had an exceptional response to the NTRK inhibitor, larotrectinib^7^. In contrast, one can posit that the lack of response to NTRK inhibition in our case might be explained by the presence of the *NBN* LOF mutation and genomic instability that has been associated with resistance to targeted therapies^20,28^.

Taken together, our findings are consistent with *NBN* LOF having played a role in the progression of this PACC, and provide evidence that LOF of DDR/HR-related genes may have a causative role in PACC more broadly. This observation warrants the consideration of other HR/DDR genes in ongoing studies of PARP inhibitors in pancreatic cancers, including PACC (NCT04858334 and NCT05286827). Finally, our report highlights the unique insights and the clinical impact stemming from comprehensive genomic analysis of rare tumors, such as PACC.

## Methods

The patient reported in this case study was enrolled in a monotherapy arm of TRESR (NCT04497116), an ongoing, modular, phase 1/2a, first-in-human, multicenter, open-label, non-randomized, dose-escalation, dose-expansion study of camonsertib administered orally as a single agent, or in combination with talazoparib or gemcitabine in patients with advanced solid tumors. The study was conducted in accordance with the Declaration of Helsinki and Council for International Organizations of Medical Sciences International Ethical Guidelines, applicable International Conference on Harmonization Good Clinical Practice Guidelines, and applicable laws and regulations. The patient provided written informed consent which allowed for in-depth genomic investigation of the tumor and normal tissue specimens provided. The protocol was approved by the Institutional Review Board or ethics committee at the treating institution, Sarah Cannon Research Institute/Tennessee Oncology.

### Tissue specimen review

Formalin-fixed paraffin-embedded (FFPE) tissue specimens corresponding to the primary resection (November 2018) and metastatic liver biopsy (July 2021) were retrieved. After biopsy, tissue specimens were immediately fixed in 10% neutral buffered formalin for 12–24 h. Genomic DNA and total RNA were extracted (Invitae; San Francisco, CA) using the AllPrep DNA/RNA FFPE kit (Qiagen, cat# 80234) and quantified using the Quant-iT dsDNA Assay Kit, broad range (ThermoFisher, cat# Q33120) at NeoGenomics using documented standard operating procedures as part of conduct of the trial (Supplementary Table 1). Corresponding hematoxylin and eosin sections were reviewed by a board-certified pathologist to review tumor cellularity and histology to confirm diagnoses. BCL-10 staining of the screening tissue to confirm the diagnosis was performed at NeoGenomics.

### Biomarker monitoring

Serum CA19-9 levels were monitored routinely via commercial laboratory testing.

### Clinical-grade NGS

Prior to enrollment into TRESR (NCT04497116), the patient’s tumor tissue specimens were analyzed by Tempus xT and Foundation Medicine FoundationOne CDx to guide therapy selection as part of standard of care at the treating institution^16,19^.

### Retrospective central NGS testing

#### SNiPDx™ targeted sequencing

SNiPDx™ is a novel targeted sequencing panel capable of distinguishing monoallelic and biallelic LOF alterations in select DNA damage repair genes^15^. DNA (minimum of 30 ng) was extracted from 10 × 5 µm FFPE slides. DNA was analyzed on a custom AMP panel comprising 26 genes, referred to as SNiPDx*™* ^15^. Libraries were quantitated using quantitative PCR (qPCR; Kapa Biosystems, Woburn, MA, USA) per manufacturer protocol. Amplicon sequencing was performed on the NovaSeq platform (Illumina, San Diego, CA, USA) according to the manufacturer’s standard protocol. Paired-end sequence data were processed using methods developed for AMP to align error-corrected reads^18^. AMP libraries were processed using the VariantPlex Pipeline from Archer Analysis Platform v6.2.8.

Genome-wide major and minor copy numbers were inferred by FACETS^29^. Briefly, copy number alterations and allelic imbalances in the 26 SNiPDx™ target genes and other genomic regions were calculated on the basis of the Log_2_R (ie, the Log_2_ ratio of single nucleotide polymorphism coverage in a tumor specimen to coverage in a matched panels of normal), and Log_2_ odds ratio (Log_2_OR; calculated from the number of reads reporting the alternative allele:number of reads reporting the reference allele), adjusted by tumor purity and ploidy.

#### Whole genome sequencing

All FFPE-isolated DNA was quantified by KAPA SYBR FAST qPCR quantification and quality control (QC) kit (Kapa Biosystems, Woburn, MA, USA). QC criteria required a minimum DNA concentration of >1 ng/µL and minimum amplifiable ratio of >0.1. WGS was performed on the NovaSeq targeting 60x coverage for tumor and 30x coverage for PBMCs. Genome sequencing covered a minimum of 95% of the genome with 20x coverage or higher. Nucleic acid from the submitted specimen was isolated and the library products were sequenced with 2 × 150 base pair reads using the Illumina NovaSeq sequencing instrument (Illumina, San Diego, CA). After alignment to the reference genome (GRCh37/hg19), off-target, low quality, and duplicate reads were removed from the analysis. The targeted regions were assessed for average depth of coverage and other data quality thresholds.

WGS analysis was performed as previously described^20,21^. Raw sequencing reads from FASTQ were aligned using the Burrow-Wheelers Aligner^30^ to the GRCh37/hg19 reference. SNVs were called using MuTect^31^. Short indels were called using VarScan2^32^, Strelka^33^, Platypus^34^, and Scalpel^35^. Allele-specific copy number analysis was performed using FACETS^29^. Mutational signatures were decomposed using SIG NAL^36^. Structural variants were called using Manta^37^ and GRIDSS^38^. HRD scores were calculated using HRDetect^23^.

#### Targeted RNA sequencing

Targeted RNA sequencing was performed utilizing the Archer^®^ FusionPlex^®^ Custom Solid Panel with Anchored Multiplex PCR (AMP™; Invitae) at the Memorial Sloan Kettering Integrated Genomics Operation as previously described^17,39^. Results were analyzed utilizing the FusionPlex^®^ standard analytical suite as previously described^17,40^.

### Reporting summary

Further information on research design is available in the Nature Research Reporting Summary linked to this article.

### Supplementary information

Supplementary Material

REPORTING SUMMARY

## Data Availability

DNA and RNA sequencing data from this study are not publicly available because they contain potentially identifiable information on the patient, and there is no consent to deposit the data into a repository. For eligible studies, qualified researchers may request access to individual patient-level clinical data through a data request platform. At the time of writing, this request platform is Vivli (https://vivli.org/ourmember/roche/). Datasets may be requested 18 months after a clinical study report has been completed and, as appropriate, once the regulatory review of the indication or drug has completed. Access to patient-level data from this trial may be requested and will be assessed by an independent review panel, which decides whether the data will be provided, taking the risk of patient re-identification into consideration. Once approved, the data are available for up to 24 months. Anonymized records for individual patients across more than one data source external to Roche cannot, and should not, be linked owing to a potential increase in risk of patient re-identification. For up-to-date details on Roche’s Global Policy on the Sharing of Clinical Information and how to request access to related clinical study documents, see https://go.roche.com/data_sharing.

## Code availability

The analysis of SNiPDx™ data is based on R package FACETS, where version 0.6.1 was used, downloaded from URL: https://github.com/mskcc/facets.
