# Supplementary material for: Post-therapy emergence of an NBN reversion mutation in a patient with pancreatic acinar cell carcinoma
Source: NPJ Precis Oncol. 2024 Apr 1;8:82. doi: 10.1038/s41698-024-00497-x (PMC10985087; doi:10.1038/s41698-024-00497-x)
Supplement: Supplementary file 1 — Supplementary Material [file 41698_2024_497_MOESM1_ESM.pdf]

SUPPLEMENTARY MATERIAL

Supplementary Table 1: Tumor content and DNA extraction yield

Specimen	Fixation time	Tumor cell content (Path)	Extracted DNA (ng)	Extracted RNA (ng)	Tumor cell content (SNiPDX)	Tumor cell content (WGS)
Primary resection	Immediate post biopsy, then 12–24 hours	90%	16,948	22,500	33%	N/A
Metastatic biopsy	Immediate post biopsy, then 12–24 hours	50%	4,208	12,210	56%	66%

SNIPDx, SyNthetic lethal Interactions for Precision Diagnostics; WGS, whole-genome sequencing.

Supplementary Figure 1. B allele frequency/log R ratio plots derived from SNiPDx™ analysis of a) the primary resection and b) the metastatic liver biopsy.

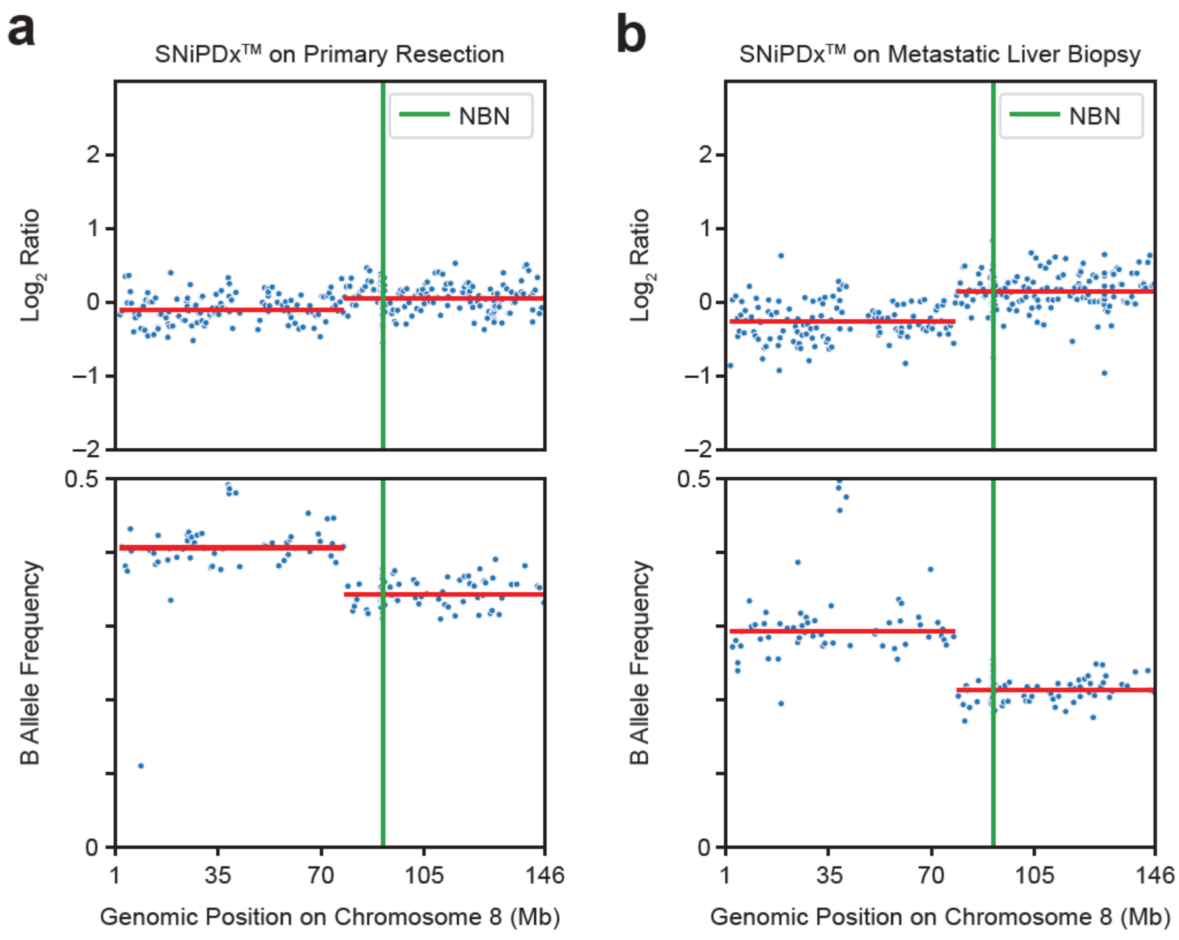

SNiPDx, SyNthetic lethal Interactions for Precision Diagnostics; Mb, megabase.
