# Supplementary material for: Post-therapy emergence of an NBN reversion mutation in a patient with pancreatic acinar cell carcinoma
Source: NPJ Precis Oncol. 2024 Apr 1;8:82. doi: 10.1038/s41698-024-00497-x (PMC10985087; doi:10.1038/s41698-024-00497-x)
Supplement: Supplementary file 2 — REPORTING SUMMARY [file 41698_2024_497_MOESM2_ESM.pdf]

nature portfolio

Corresponding author(s): Meredith S. Pelster, MD, MSCI

Last updated by author(s): Oct 13, 2023

Reporting Summary

Nature Portfolio wishes to improve the reproducibility of the work that we publish. This form provides structure for consistency and transparency in reporting. For further information on Nature Portfolio policies, see our Editorial Policies and the Editorial Policy Checklist.

Statistics

For all statistical analyses, confirm that the following items are present in the figure legend, table legend, main text, or Methods section.

n/a	Confirmed	
☒	☐	The exact sample size (n) for each experimental group/condition, given as a discrete number and unit of measurement
☒	☐	A statement on whether measurements were taken from distinct samples or whether the same sample was measured repeatedly
☒	☐	The statistical test(s) used AND whether they are one- or two-sided<br>*Only common tests should be described solely by name; describe more complex techniques in the Methods section.*
☒	☐	A description of all covariates tested
☒	☐	A description of any assumptions or corrections, such as tests of normality and adjustment for multiple comparisons
☒	☐	A full description of the statistical parameters including central tendency (e.g. means) or other basic estimates (e.g. regression coefficient) AND variation (e.g. standard deviation) or associated estimates of uncertainty (e.g. confidence intervals)
☒	☐	For null hypothesis testing, the test statistic (e.g. F, t, r) with confidence intervals, effect sizes, degrees of freedom and P value noted<br>*Give P values as exact values whenever suitable.*
☒	☐	For Bayesian analysis, information on the choice of priors and Markov chain Monte Carlo settings
☒	☐	For hierarchical and complex designs, identification of the appropriate level for tests and full reporting of outcomes
☒	☐	Estimates of effect sizes (e.g. Cohen's d, Pearson's r), indicating how they were calculated

Our web collection on statistics for biologists contains articles on many of the points above.

Software and code

Policy information about availability of computer code

Data collection	No software was used for data collection
Data analysis	SNiPDx analysis - R package FACETS version 0.6.1; WGS analysis - Burrow-Wheelers Aligner, MuTect, VarScan2, Strelka, Platypus, Scalpel, FACETS, SIGNAL, Manta, GRIDSS, HRDetect

For manuscripts utilizing custom algorithms or software that are central to the research but not yet described in published literature, software must be made available to editors and reviewers. We strongly encourage code deposition in a community repository (e.g. GitHub). See the Nature Portfolio guidelines for submitting code & software for further information.

Data

Policy information about availability of data

All manuscripts must include a data availability statement. This statement should provide the following information, where applicable:

- Accession codes, unique identifiers, or web links for publicly available datasets
- A description of any restrictions on data availability
- For clinical datasets or third party data, please ensure that the statement adheres to our policy

DNA and RNA sequencing data from this study are not publicly available because they contain potentially identifiable information on the patient, and there is no consent to deposit the data into a repository. For eligible studies, qualified researchers may request access to individual patient-level clinical data through a data request platform. At the time of writing, this request platform is Vivli (https://vivli.org/ourmember/roche/). Datasets may be requested 18 months after a clinical

study report has been completed and, as appropriate, once the regulatory review of the indication or drug has completed. Access to patient-level data from this trial may be requested and will be assessed by an independent review panel, which decides whether the data will be provided, taking the risk of patient re-identification into consideration. Once approved, the data are available for up to 24 months. Anonymized records for individual patients across more than one data source external to Roche cannot, and should not, be linked owing to a potential increase in risk of patient re-identification. For up-to-date details on Roche's Global Policy on the Sharing of Clinical Information and how to request access to related clinical study documents, see https://go.roche.com/data_sharing.

Human research participants

Policy information about studies involving human research participants and Sex and Gender in Research.

Reporting on sex and gender	N/A- case report
Population characteristics	N/A- case report
Recruitment	Patient was selected based on enrollment criteria for the TRESR trial
Ethics oversight	IRBs or ethics committees at trial sites

Note that full information on the approval of the study protocol must also be provided in the manuscript.

Field-specific reporting

Please select the one below that is the best fit for your research. If you are not sure, read the appropriate sections before making your selection.

☒ Life sciences ☐ Behavioural & social sciences ☐ Ecological, evolutionary & environmental sciences

For a reference copy of the document with all sections, see nature.com/documents/nr-reporting-summary-flat.pdf

Life sciences study design

All studies must disclose on these points even when the disclosure is negative.

Sample size	N/A - case report
Data exclusions	N/A
Replication	Human tissue and blood were analyzed in single replicates due to sample availability
Randomization	N/A
Blinding	N/A

Reporting for specific materials, systems and methods

We require information from authors about some types of materials, experimental systems and methods used in many studies. Here, indicate whether each material, system or method listed is relevant to your study. If you are not sure if a list item applies to your research, read the appropriate section before selecting a response.

Materials & experimental systems

n/a	Involved in the study	
☒	☐	Antibodies
☒	☐	Eukaryotic cell lines
☒	☐	Palaeontology and archaeology
☒	☐	Animals and other organisms
☐	☒	Clinical data
☒	☐	Dual use research of concern

Methods

n/a	Involved in the study	
☒	☐	ChIP-seq
☒	☐	Flow cytometry
☒	☐	MRI-based neuroimaging

Clinical data

Policy information about clinical studies

All manuscripts should comply with the ICMJE guidelines for publication of clinical research and a completed CONSORT checklist must be included with all submissions.

Clinical trial registration	NCT04497116
Study protocol	Attached as part of the submission
Data collection	Data was collected at the treating institution, Tennessee Oncology, from December 2017 through March 2022; patient was enrolled in TRESR in July 2021.
Outcomes	See attached protocol
